# Envisioning a path from the Internet of Medical Things to improved fertility care access: a mini-review

**DOI:** 10.1016/j.xfre.2024.09.004

**Published:** 2024-09-26

**Authors:** Olutunmike Kuyoro, Randi Goldman

**Affiliations:** Northwell, New Hyde Park, New York; Department of Obstetrics and Gynecology, Northwell Health Fertility, New York, New York; Zucker School of Medicine at Hofstra, Uniondale, New York

**Keywords:** Internet of Things, ART, fertility care access, wearable devices, blockchain

## Abstract

Devices that function within a network of interconnected systems and are equipped with sensors, software, and tools designed to collect and exchange data are widely known as the Internet of Things (IoT). In recent years, the rapid growth of IoT technology has sparked significant interest in leveraging these systems to enhance healthcare delivery across various medical fields, including fertility care and assisted reproductive technology. The subset of IoT devices applied within the healthcare sector is referred to as the Internet of Medical Things (IoMT). Despite this growing technological potential, there has been little exploration into using IoMT to address a critical challenge in fertility care: the ongoing lack of access for many individuals in need of these services. In this article, we examine various applications of IoMT in healthcare, as well as how these may be extrapolated to the fertility field, and more importantly, assess how they may aid in bridging the access gap. We also explore potential challenges and pitfalls associated with implementing the IoMT, which underscores the need for careful oversight in its adoption and utilization.

The Internet of Things (IoT), first described over two decades ago, refers to the network of physical objects equipped with sensors, software, and technologies developed with the aim of connecting and exchanging data with other devices. The Internet of Medical Things (IoMT) is a subset of the IoT, focusing on the application of connected devices in the healthcare and medical sectors, which theoretically could provide new avenues of improved healthcare delivery in a vast range of medical fields. Since its inception over a decade ago, there has been an exponential growth of IoT devices, and as of 2023, there were 16.7 billion active endpoints (1).

Although infertility is one of the most common diseases among reproductive age populations, access to treatment remains elusive for a large number of individuals who struggle with the condition (2). In the fertility field, IoMT is currently being investigated as a tool for use in all aspects of patient care, from sperm and oocyte identification, to embryo selection and optimizing in vitro fertilization protocols (3). However, literature is lacking as to how advancements in any of these areas may facilitate systems-based improvement in one of the major aspects of fertility care that continues to lag behind others—access. Previous studies have demonstrated that the leveraging of IoMT technology can help lower healthcare costs for both patients and healthcare systems, which is often a key barrier to access. For example, the use of an ambulatory remote monitoring system, comprised of a mobile app and linked telemonitoring smart devices, significantly reduced hospital readmission rates and healthcare expenses among patients with heart failure (4).

## Applications of iomt in fertility care

Various facets of IoMT have been integrated into healthcare with the purported aims of improving patient outcomes and healthcare access, increasing efficiency, and reducing costs. The degree to which their current iterations have succeeded in achieving these aims is discussed.

### Wearable menstruation and fertility tracking devices

There now exists several technological advances developed to enable individuals self-monitor physiological parameters associated with the hormonal fluctuations that occur throughout the menstrual cycle, and although the majority of these technologies began in the form of commercially available applications for smartphones, the paradigm appears to be shifting to the use of wearable devices. The global market for these fertility trackers is estimated to be worth approximately USD 16.18 billion and is projected to exceed a valuation of USD 44.72 billion by 2032 (5).

The theoretical advantage of wearable devices over mobile apps is that rather than relying on data manually entered by the user, which may be subject to recall errors, these devices continuously collect and interpret objective data from physiological parameters such as heart rate, body temperature, and sleep cycle changes. These data are tracked to offer users clear, understandable insights about the stages of their menstrual cycle. Although using indicators such as heart rate and sleep patterns is relatively new, and no studies have directly compared their effectiveness against traditional markers like body temperature alone, strong correlations have been established between these metrics and different phases of the menstrual cycle (5, 6). For instance, research shows a consistent increase in heart rate from ovulation through the luteal phase, with a peak during the mid-luteal phase. Additionally, the luteal phase has been linked to both shorter overall sleep duration and reduced rapid eye movement sleep.

Examples of popular commercially available wearable devices are the Oura ring, the Femometer smart ring, and the Ava bracelet (Table 1). In terms of improving healthcare access, these devices may contribute toward this goal by democratizing health information and empowering individuals with data about their own menstrual cycles, ovulation periods, and fertility windows. Such information may be especially invaluable for geographically underserved populations in which there is limited access to reproductive health specialists. Moreover, with the potential to integrate with telehealth services, users may be able to share data with healthcare providers remotely and potentially reduce the frequency of in person visits. With the advantage of being able to consistently monitor fertility-related markers, these devices may aid in the early detection of irregularities such as abnormal menstrual cycles or ovulation patterns. Early detection could engender timely consultation and intervention, potentially identifying underlying health issues that could negatively affect fertility. This may be especially beneficial for ethnic minorities who have been shown to initiate fertility care at significantly older ages than their White counterparts (6, 7).

**Table 1:** Currently available wearable fertility trackers.

Wearable technology	Parameter measured	Reported ovulation prediction accuracy	Costs	FDA approval
Oura ring	HR, HRV, and skin temperature	93%–98%	Initial: $300–$500 Recurrent: Monthly $6 subscription fee	Yes
Femometer smart ring	Skin temperature	99%	Initial: $149 Recurrent: N/A	No
OvulaRing	Vaginal temperature	—	Initial: $400–$1100 Recurrent: N/A	No
Ava wrist device	HR, RR, skin temperature, skin perfusion, movement	90%	Initial: $249 Recurrent: N/A	Yes
Tempdrop armband tracker	Axillary temperature	—	Initial: $169–$219 Recurrent: N/A	No

*Note:* FDA = Food and Drug Administration; HR = heart rate; HRV = heart rate variation; RR = respiratory rate.

A contentious aspect of the ability of these devices to improve fertility care accessibility is the costs involved. Although an argument may be made for long-term cost reduction that may be achieved by eliminating the need for more frequent in person visits or invasive monitoring, some of these devices have initial purchase prices as well as ongoing costs that may prove prohibitive to some patients who already significant financial constrains (Table 1).

Most research today has focused on the mobile applications that have been shown with some frequency to be of limited accuracy (8); however, there are a handful of studies that have attempted to assess the validity of these wearable devices. A systematic review of these studies conducted by Lyzwinski et al. (9) aimed to evaluate the effectiveness of these devices at tracking the various menstrual cycle stages. Their findings revealed that although the reported accuracy, sensitivity, and specificity of these devices for fertility and menstrual cycle prediction appear encouraging, the small number of participants included in these studies means that caution should be exercised when interpreting these results. Moreover, in addition to small sample sizes, another limitation of many studies included in the systematic review was that they were single-armed pilot studies. Better quality comparative, prospective studies with larger sample sizes are needed to compare commercially available wearables with other methods of menstrual cycle tracking, particularly as they relate to narrowing the fertility access gap. Until then, there likely will remain a lack of much needed deliberation and engagement in the development, assessment, and regulation of these wearable devices.

### At-home fertility testing devices

In addition to wearable devices that track physiological parameters to provide information on the menstrual cycle, there are self-powered IoMT-linked diagnostic devices that have been developed to provide real-time evaluation of biomarkers in and on the human body such as urine, blood, sperm, and sweat.

In the fertility field, such devices have been harnessed specifically for at-home male and female fertility testing. These devices are often relatively inexpensive, user-friendly smartphone-linked devices, which claim to accurately measure markers of fertility in male and female patients. These devices may represent fast, convenient, and cost-effective way of identifying patients with subfertility, and thus a step forward in bridging the access gap. Supporting this notion are results from questionnaire studies that showed that participants preferred and were more likely to use at-home self-semen analysis rather than standard laboratory testing (10, 11). This is particularly relevant to efforts toward closing the access gap, as ethnic minorities have been shown to be more likely to delay seeking care and evaluation for fertility issues (12).

For male patients, the ability to perform semen analysis at-home may offer a way of overcoming the issue of limited male partner involvement in the fertility workup and thus increase likelihood of follow-up. In general, the smartphone-based semen analysis tests work by coupling the inbuilt phone camera with an external optical attachment that enables image magnification for semen analysis. For example, the Food and Drug Administration approved YO semen analysis test uses the phone’s camera and light source attached to a mini microscope to capture a recording of sperm (13). Its proprietary software then analyzes the light fluctuations caused by motile sperm in the recording and converts these readings into motile sperm concentration. The accuracy of this device and others (Table 2) has been evaluated in several studies (10, 11, 14), most of which have concluded that there is a high degree of concordance between results obtained by these at-home testing systems and standard laboratory techniques.

**Table 2:** Currently available at-home fertility testing devices.

Device	Technique	Results yielded	Time to yield result (min)	Reported accuracy	Cost	FDA approval
YO sperm clip	Smartphone-based device with optical tracking	Concentration Motile sperm	15	Smartphone type dependent (97%–98%)	$79.95	Yes
ExSeed	Smartphone-based device with optical tracking	Semen volume Concentration Motility Total motile sperm count	15	95%	$84.99	No
SEEM	Smartphone-based device with optical tracking	Volume Concentration Motility	15	96%	$45	Yes
Kegg	Smartphone-linked vaginal device	Cervical mucus quality	2–3	–	$259	No
Proov Complete Testing System	Smartphone-linked urinary testing strips	FSH LH Progesterone Estrogen	10	99%	$94.99	Yes
Mira Hormone Monitor Max Kit	Smartphone-linked urinary testing wands	FSH LH Progesterone Estrogen	21	99%	$229	Yes

*Note:* FDA = Food and Drug Administration; FSH = follicle-stimulating hormone; LH = luteinizing hormone.

A limitation to the use of these devices is the paucity of semen parameters that can be evaluated by each device, with none of the currently available devices being capable of measuring all the World Health Organization recommended parameters for semen analysis. Furthermore, as noted by Drobnis and Rothmann (15), although semen analysis represents only part of the fertility workup, a supposedly normal semen analysis result may actually impede the individual from seeking further care and thus prevent identification of other potential etiologies of infertility in the male patient. It is for this reason these investigators caution against the widespread adoption of this method of semen analysis as a substitute for laboratory testing before more vigorous validation studies have been conducted.

At-home female fertility testing devices, on the other hand, run the gamut from devices that test cervical fluid to those that test urine hormone levels (Table 2), all developed to make inferences about fertility status. However, at present, there are no commercially available serum-based IoMT-linked fertility testing devices. The urinary hormone level testing devices typically employ lateral flow assay test strips paired with smartphone applications to provide information about the fertility window. Examples include the Proov Complete and the Mira Hormone Monitor Kit both of that are Food and Drug Administration approved. The only commercially available IoMT-linked cervical mucus monitoring device is the Kegg device that has been shown to be superior to the use of basal body temperature to predict ovulation (16). It works with the use of electrical impedance spectroscopy to monitor cervical mucus electrolyte trends to confirm a fertility window.

Although the manufacturer-reported accuracies for these devices appear to be relatively high (Table 2), externally validated data are reported by only a handful of studies (17, 18, 19). Therefore, it is important to exercise caution before attributing these devices with the ability to significantly expand access.

### Blockchain, federated learning, and the decentralization of “big data”

Blockchain is a distributed ledger technology that allows data (including that collected by IoMT devices) to be stored across a network of computers in a manner that is theoretically secure, transparent, and tamper resistance. Unlike traditional databases that are stored in a single central server or location, distributed ledger technology is decentralized and distributed across multiple locations, allowing multiple parties to possess copies of the same ledger. This allows for transparency and consensus. Storing data using the chaining mechanism results in an essentially immutable data set, meaning that any effort to change the established blocks is readily apparent. The IoMT in theory could allow for the collection, analyses, and linkage of large volumes of data that can shed light on population-level disease patterns and identify risk factors that ultimately may aid in enhancing the effectiveness, efficiency, and consistency of clinical decisions made during fertility treatment. With such improvements, one could imagine a reduction in the disparities in fertility care access and outcomes we have today. In addition to the large amounts of data that would be required to make associations between factors and outcomes, crucial to the use of such data would be the collection of data points from diverse sources that would allow for generalizability. Although there are over 2 million assisted reproductive technology (ART) cycles performed each year worldwide, and thus collection of such data should be an ostensibly attainable goal, this however remains an elusive objective for many data gathering tools that exist today (20). At present, there is no system in place to adeptly integrate the pertinent ART data. The scarcity of mandatory registries globally, as well as the paucity of detail within the registries that do exist has meant that researchers face considerable challenges in obtaining data of high enough quality to develop algorithms—an indispensable requirement for devising the appropriate predictions that can aid in sound clinical decision making (20).

Enter the blockchain. With a blockchain system of data sharing in place, the inter-clinic variabilities that currently preclude the conduction of large prospective studies could be reduced and hence hasten the pace of output of results from studies that drive the fertility field forward.

**Figure 1:**
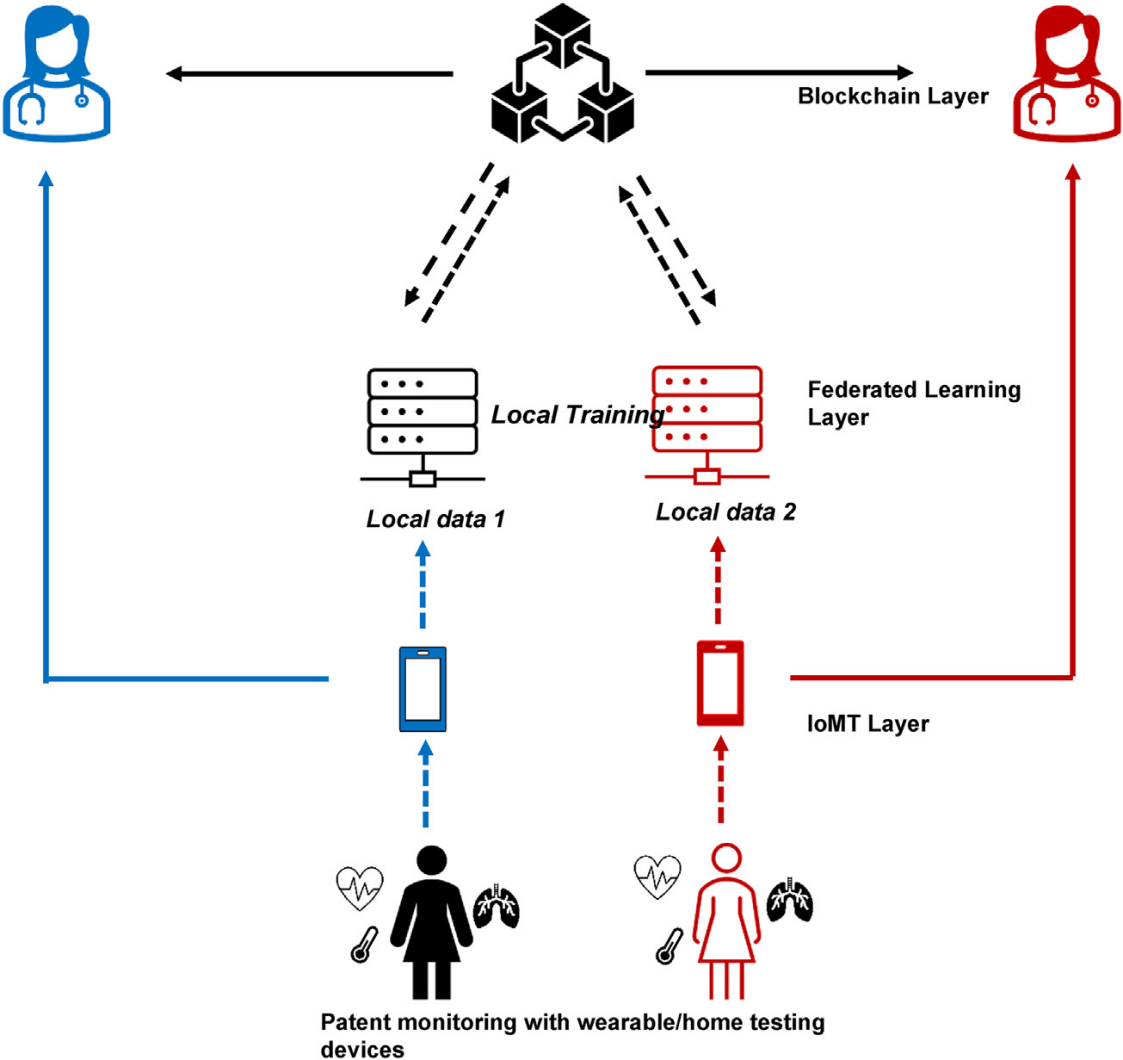
IoMT-federated learning-blockchain architecture. IoMT = Internet of Medical Things.

The other facet of “big data”-driven innovation that could be incorporated into fertility care is federated learning. Healthcare data are often very sensitive and thus require stringent guidelines put in place to maintain patient privacy and confidentiality, as well as a means of tracking interactions with this sensitive data. Federated learning is a decentralized approach to machine learning, aimed at addressing the issue of data governance and privacy wherein multiple devices and servers collaboratively train models without sharing raw data (20). The result is a three-layered architecture wherein the IoMT devices collect data, the machine learning processes are applied through federation learning for privacy safeguarding purposes, and the blockchain layer is responsible for aggregation of the gathered data (Fig. 1).

Used in tandem, blockchain technology and federated learning may serve as the foundation for a data sharing platform that has wide ranging applications in the fertility field including but not limited to gamete and embryo donation matching, the execution of multi-part legal contracts, signing of patient consents, and tracking data pertaining to transport and storage of gametes and embryos (20). By streamlining these processes and therefore improving efficiency, the use of blockchain could potentially reduce overhead costs and administrative burdens, making fertility services more affordable and accessible to patients for whom it was previously not.

Although current applications of these technologies in healthcare remain in their infancy or research stage, there are a few promising developments in several different healthcare fields. One such development is in the medical imaging field, where the use of blockchain as a distributed data store has been used to develop a framework for image sharing, aimed at improving the already existing Image Sharing Network developed by the Radiological Society of America (21). In contrast to the Image Sharing Network, the blockchain ledger of radiological studies created by Patel (21) eliminated third-party access to protected health information and allowed patients to access to their medical images in a timely and secure fashion. This ledger could serve as a foundation for developing similar systems that would enable patients to securely and easily transfer other types of health information, such as laboratory results, across different platforms.

## Barriers to adoption of iomt

In addition to some of these concerns discussed, there are key challenges to IoMT that must be addressed before widespread adoption.

### Privacy and security risks

Although the interconnected nature of IoMT devices allows for increased data gathering capabilities, it also makes them potential targets for cyberattacks and inappropriate collection of personal and sensitive patient data. Additionally, as healthcare data become a commodity, it could allow for the marketing of patient health information without appropriate consents. To mitigate these risks, healthcare organizations must set as a priority the development of cybersecurity measures to protect from breaches and unauthorized access. The use of blockchain that employs robust cryptographic algorithms to create tamper proof records of all data transactions may help to ensure that patient health information remains unchanged and maintains its integrity. Organizations must also ensure that they regularly assess the risks and weaknesses of their data management systems and implement robust interference detections systems and security monitoring tools to detect and respond to any unauthorized access.

### Scalability

Scalability refers to the ability of a healthcare device to adapt to changes to its environment, with highly scalable systems being ones that maintain conformity among their interconnected devices and thus remain usable in current and future time points. Many existing blockchain structures struggle to handle the ever-increasing volume and speed of healthcare data transactions especially in healthcare systems that generate vast amounts of data (20). Further research is undoubtedly needed to develop innovative methods of improving scalability of blockchain in ART-specific healthcare systems.

### Standardization and interoperability

Because IoMT devices and systems are developed by different manufacturers that likely use different proprietary systems, interoperability remains a significant challenge because data exchange between the different systems becomes increasingly unwieldy that results in the creation of what has been termed vertical silos (22). To ameliorate this particular constraint, standardized or more homogenous interfaces are required to allow for more seamless data exchange and integration of IoMT devices and systems across different healthcare settings and to provide a more satisfying user experience for both patients and clinicians. It should also be noted that although the creation of blockchains does not require standardized databases, the standardization of data formats within the blockchains themselves is important, and agreement on a standardized format between the stakeholders of a blockchain can enhance interoperability, data exchange, and integration with existing systems.

### Impact on healthcare workers

The impact of IoMT of healthcare workers is a multifaceted one. Although potentially allowing for increased efficiency and productivity, the inevitable automation of many jobs raises concerns regarding the possibility of job displacement for healthcare workers. It is therefore imperative that healthcare organizations invest in upskilling and reskilling initiatives for employees (23).

## Conclusion

Although IoMT certainly has the potential to transform the way fertility care is delivered, from remote monitoring to enhanced data collection capabilities, its success in alleviating healthcare disparities is contingent on the mitigation of challenges such as bolstering data privacy and security, improving interoperability of different devices, and ensuring that both healthcare providers and patients are equipped with the requisite training and resources to wield these technologies effectively.

## CRediT Authorship Contribution Statement

**Olutunmike Kuyoro:** Conceptualization, Writing - original draft, Writing - review & editing. **Randi Goldman:** Supervision, Writing - review & editing.

## Declaration of Interests

O.K. has nothing to disclose. R.G. reports consulting fees from UpToDate as a peer reviewer, outside the submitted work.
